# Millennials’ Consumption of and Attitudes toward Meat and Plant-Based Meat Alternatives by Consumer Segment in Finland

**DOI:** 10.3390/foods11030456

**Published:** 2022-02-03

**Authors:** Antti Knaapila, Fabienne Michel, Kirsi Jouppila, Tuula Sontag-Strohm, Vieno Piironen

**Affiliations:** 1 Department of Food and Nutrition, University of Helsinki, P.O. Box 66, FI-00014 Helsinki, Finland; kirsi.jouppila@helsinki.fi (K.J.); tuula.sontag-strohm@helsinki.fi (T.S.-S.); vieno.piironen@helsinki.fi (V.P.); 2 Department of Health Sciences and Technology, ETH Zürich, Universitätstrasse 16, 8092 Zürich, Switzerland; fabienne.michel@alumni.ethz.ch

**Keywords:** acceptance, consumer segmentation, flexitarian, meat analogue, meat substitute, online survey, plant-based protein, sustainability, vegan, vegetarian

## Abstract

Millennials are considered the key generation with regard to the consumption of plant-based meat alternatives via flexitarianism. This study sought to characterize millennials’ consumer segments based on their consumption of and attitudes toward meat and meat alternatives. We conducted an online survey on the hedonic tones of the associations evoked by meat and meat alternatives, consumption of such foods, and diet-related attitudes among a representative sample of Finnish millennials (*N* = 546, 59% women, age 20–39 years). Some 41% of respondents regularly ate plant-based meat alternatives, while 43% had tried such foods. We divided the respondents into six segments based on the hedonic tones of their meat vs. meat alternatives associations. The segments differed in terms of their consumption of meat alternatives and the underlying reasons why, importance of meat in meals, and Meat Commitment Scale scores. The segment that reported much more positive associations with meat than meat alternatives (~14% of the respondents) may prove resistant to interventions intended to reduce meat intake, whereas the segment that displayed the most positive attitudes toward meat alternatives (~18%) did not eat much meat. Thus, the four middle segments (totaling ~68%), whose associations’ hedonic tones were close to each other, may be the best targets for future interventions designed to reduce meat consumption through the use of meat alternatives. To conclude, introducing a simple segmentation allowed us to identify consumer segments with large potential to reduce meat consumption.

## 1. Introduction

The need for more environmentally sustainable alternatives to meat (and especially to red and processed meat) is increasing due to planetary boundaries (i.e., global biophysical limits for safe operating space in, e.g., climate change, biosphere integrity, land-system change, and freshwater use [1]) limiting the capacity to produce more meat for the increasing global population [2]. In addition, while meat is an important source of nutrients, especially protein, heavy meat consumption may have adverse effects on human health (for a review, see [3]). The EAT–Lancet Commission on Healthy Diets from Sustainable Food Systems stated that the “transformation to healthy diets by 2050 will require substantial dietary shifts, including a greater than 50% reduction in global consumption of unhealthy foods, such as red meat and sugar” [2]. This goal will likely prove difficult to achieve, as global meat consumption (both the average per capita and total consumption) continues to rise [3].

Food products that are made of protein-rich nonanimal sources intended to resemble meat and that are used instead of meat are often referred to as meat analog(ue)s, meat substitutes, or meat alternatives. In the literature, these terms are generally used synonymously [4], although their definitions do sometimes differ among authors. The term meat analogue has been commonly used in recent reports on the production of such products using extrusion technology [5,6,7,8,9,10,11]. For instance, Kumar et al. [12] defined a meat analogue as “a food product that approximates the aesthetic qualities and/or chemical characteristics of certain types of meat. These are made from non-animal protein and their appearance and smell are very much similar to meat”. Dekkers et al. [13] considered functionality alongside sensory properties and defined meat analogues as “products that can replace meat in its functionality, being similar in product properties/sensory attributes, and that can also be prepared by consumers as if they were meat”. Moreover, the terms meat analogues and meat substitutes are often used to refer to products that more closely resemble meat in terms of their sensory properties than meat alternatives, a term that is used in a broader sense to refer to alternatives to meat. For example, Elzerman et al. [14] defined meat substitutes as “products that were developed to be eaten instead of meat” (e.g., vegetarian sausages and steaks) and meat alternatives as “other products that are often eaten as protein source in vegetarian meals, such as pulses and nuts”. However, Choudhury et al. [15] regarded plant-based meat alternatives as a “sustainable source of proteins that can match the taste and texture, color, and nutritional profile of specific types of meat”. Based on the previously mentioned studies, it appears that a consensus has not yet been reached concerning the terminology for these products.

Meat intake can be reduced in many ways and with proteins derived from many sources: using conventional vegetarian foods (e.g., pulses), hybrid meat products (containing both meat and plant-based ingredients) [16], and meat alternatives. The most commonly used alternative protein ingredients originate from plants (especially soy, pea and other legumes, oilseeds, and wheat), fungi (mycoprotein), insects, and algae (macroalgae and microalgae) [4,17,18]. In addition, cultured meat (in vitro meat) is regarded as an alternative to meat from livestock [4,17]. To distinguish among the different protein sources and so render the utilized term more precise, the source of the protein is sometimes included, for example, in plant-based meat alternatives. This term has been used to refer to commercial products in several recent reports, including some consumer studies [4,15,19,20,21,22,23]. Likewise, we used the term plant-based meat alternatives in the present study because it focused on respondents’ orientations specifically toward plant-based alternatives to meat.

Plant-based proteins appear to be the most widely accepted meat alternatives/alternative proteins from the perspective of consumers [23,24]. Gómez-Luciano et al. [25] investigated the willingness to purchase three types of meat alternatives (plant-based proteins, cultured meat, and insects) on the part of consumers from four countries with dissimilar economic developmental statuses (the United Kingdom, Spain, Brazil, and the Dominican Republic) and found plant-based proteins to be the most preferred option. Similarly, Lundén et al. [26] reported Finnish consumers to prefer plant-based ingredients when compared with ingredients of insect or microbial origin.

Importantly, modern meat alternatives are targeted not only toward vegans and vegetarians but also toward flexitarians [15]. According to Dagevos [27], “a flexitarian abstains from eating meat occasionally without abandoning meat totally”. He concluded that flexitarians are not a homogeneous group that follow a strict diet; rather, they represent a middle category between consumers who regularly eat meat and those who fully abstain from it [27]. In the absence of a strict definition of what flexitarian exactly means, it is understandable that Dagevos’s review found the proportion of flexitarians to vary widely across studies, ranging from 11% to 66% [27]. Regardless of this variation, the number of flexitarians is likely to be substantially higher than the number of those who totally abstain from eating meat. Indeed, vegetarians and vegans represent only a low percentage of consumers in most countries [28], accounting for ~5% of consumers in the United States (2018) [29], 2.5% in France (2018) [30], and ~2% in Finland (2017) [31]. Therefore, flexitarianism is likely to make a substantial contribution to reducing meat consumption at the population level. However, flexitarians are a heterogeneous and rarely studied group [27]. Thus, further research on both flexitarians and prospective flexitarians is required to successfully implement strategies for reducing meat consumption [32].

The millennial generation (or millennials, who are also referred to as Generation Y) are young(ish) adults who are considered to be more knowledgeable and concerned about environmental issues than older generations [33,34]. Therefore, millennials have been the target group in recent studies concerning food sustainability [35,36]. While there is no widely accepted definition of millennials, they are often considered to be people who reached adulthood during the early 21st century, that is, the people who were born during the 1980s and 1990s [37]. Millennials also represent an important consumer group because many are presently the parents of young children, and the parents’ role is essential in terms of mediating the food consumption habits of their children [38].

Meat alternatives have the potential to grow from being niche products into mainstream ones [39]. According to the Food Sector Report by Smart Protein project [40], in Europe, the sales value of plant-based food increased by ~50% from 2018 to 2020. Yet, while the sales of plant-based meat alternatives are growing rapidly, in the United States, for example, they accounted for only around 1% of the value of all retail meat sales in 2019 [15]. In 2017, based on a review of 38 articles (published in 2004–2016) concerning consumers’ sustainable protein consumption, Hartmann and Siegrist [41] concluded that consumer awareness of the environmental impacts of meat production and consumers’ willingness to reduce meat consumption were, on average, low. Nevertheless, the market for plant-based meat alternatives is evolving rapidly, and many new companies producing meat alternatives have been founded in recent years. In fact, according to Choudhury et al. [15], more than half of all companies producing meat alternatives were founded in the last 10 years [15].

Onwezen et al. [23] recently conducted a systematic review of studies on consumer acceptance of alternative proteins. They found that the main product-related motives/barriers with regard to the use of plant-based meat alternatives stemmed from ethical, environmental, health, nutritional, and sensory aspects, in addition to familiarity/previous experiences of the products. Furthermore, the main psychological factors of relevance to the acceptability of meat alternatives were consumers’ attitudes and beliefs regarding the products as well as food neophobia [23].

It is important to note, however, that the drivers and barriers concerning the use of plant-based meat alternatives are not the same for everyone, which means that an intervention that works for one consumer segment may not be effective for a different segment [42]. Therefore, it should prove useful to achieve the meaningful segmentation of consumers and then to investigate the differences among the segments.

Consumers can be classified simply based on whether or not they eat meat alternatives. Hoek et al. [43] reported that the key barriers for nonusers of meat alternatives were unfamiliarity with the products and their lower sensory attractiveness when compared with meat. To make meat alternatives more attractive to nonusers, the authors recommended improving the sensory quality and resemblance to meat, rather than highlighting ethical arguments, because such arguments only motivated heavy users of meat alternatives. The resemblance to meat was also identified as a desirable feature for meat alternatives by Michel et al. [20]. This feature appears to be especially important for light users of meat alternatives, as the desire for similarity decreased with increasing consumption frequency in the study by Hoek et al. [43].

Consumers can also be segmented by means of a multivariate data analysis of their responses to a set of questions. For instance, Lacroix and Gifford [44] identified three consumer groups using a latent profile analysis: “meat-reducers”, “moderate-hindrance meat eaters”, and “strong-hindrance meat eaters”. Furthermore, Lemken et al. [42] searched for clusters within consumer data from Germany and New Zealand using a latent class analysis and identified five clusters in each country (three clusters were common to both countries, while two were unique for each country). Recently, Götze and Brunner [45] segmented a sample of Swiss consumers into six segments via a hierarchical cluster analysis. While the consumer groups included exclusive meat-eaters and meat-avoiders, the majority were found to lie between those extreme segments. In Finland, Niva and Vainio [46] recently studied consumers’ past, current, and intended future consumption of beef, plant-based protein products, and insect-based products. Using latent class analysis they identified five clusters of consumers, two of which (totaling ~46%) were characterized by consuming both beef and plant-based protein products. The findings of these studies are in accordance with the results of Dagevos [27] and confirm the existence of a remarkable proportion of flexitarians.

The present study sought to characterize the consumption of meat and plant-based meat alternatives as well as to provide in-depth insights into the underlying motives in this regard among various consumer segments of millennials. Based on this knowledge, we further aimed to draw conclusions regarding the potential of the segments to replace meat with meat alternatives in their diet. To achieve these aims, we conducted an online survey among a representative sample of Finnish millennials. In Finland, plant-based meat alternatives are widely available in grocery stores (brands including PulledOats, Härkis, and Beanit), making it reasonable to run this survey in the country. The criteria for the different consumer segments were defined in such a way as to allow other researchers to replicate the segmentation in future studies.

## 2. Materials and Methods

### 2.1. Overview

We conducted an online survey that was jointly designed by all the authors, initially in English. The text of the survey was then translated into other languages as required to be used in Germany, Finland, France, and the United Kingdom. The first results of the survey conducted in Germany, France, and the United Kingdom have been reported by Michel et al. [47]. Here, we report results based on data collected in Finland. These data are being reported separately because in Finland we studied the millennials whereas in the other countries respondents’ age range was wider (20–69 years, [47]) and because the questionnaire used in Finland differed somewhat from the questionaries used in the other countries. More specifically, the Finnish version included most but not all the parts of the original survey (e.g., the questions featuring pictures were excluded). The English version was translated into Finnish by four of the authors, who were all native Finnish speakers (A.K., K.J, T.S-S., and V.P.), and a research assistant from the University of Helsinki.

### 2.2. Data Collection

The required data were collected from millennials who lived in Finland. For this study, we decided to define millennials as people who were aged from 20 to 39 years at the time of the data collection (i.e., born in 1980–1999). Thus, we used age as the inclusion criterion for the study.

The nationality and ethnicity of the respondents were not probed in the survey. However, we assumed that virtually all the respondents were Finnish, as the invitations to the survey were only sent to people living in Finland and the text of the survey was solely in Finnish.

We employed a market research company (Taloustutkimus Ltd., Helsinki, Finland) to conduct the data collection in order to achieve a representative sample of millennials from among the general population of Finland. The company had its own online panel of preregistered volunteers, who were regularly invited to respond to surveys. Taloustutkimus was aware of the demographics of the registered panelists and, therefore, could invite defined samples from the panel to participate in survey studies. We provided the questions and response options for our survey to the company, which then collected responses from its online panel over the course of a week (20–26 November 2019) and provided us with data from 550 individuals.

The key concept featured in the survey was “meat alternative”. However, at the time of the study, there was no established translation of this term in Finnish. We decided to translate “meat alternative” into Finnish as “kasviproteiinituote”, although the Finnish term refers to meat alternatives made solely of plant-based proteins (the Finnish words “kasvi”, “proteiini”, and “tuote” denote “plant”, “protein”, and “product”, respectively) and so excludes other kinds of meat alternatives (such as those made of microbial proteins, whey, insects, or cultured meat).

The survey included both validated scales described in the prior scientific literature and additional questions designed specifically for this study. Lists of the questions/scales from the survey and their response options are presented in Table 1 and Table 2, wherein they are grouped thematically. Table 1 includes questions related to diet and hedonic tone concerning meat and meat alternatives and their consumption, as well as drivers and barriers associated with their consumption. Table 2 contains questions derived from published scales measuring attitudes and food-related behavior. The text of the survey in Finnish is available in the (Supplementary Materials Table S1). The survey also included a few questions that were beyond the scope of the present study and, thus, are not reported here. The age and gender of the respondents were provided by Taloustutkimus from its registry.

The questions related to the first associations with meat (Q3) and meat alternatives (Q4) were presented in a randomized order for each respondent. We placed these questions at the beginning of the survey in an effort to minimize the influence of the other items on the answers. After Q3 and Q4, we provided a definition of meat alternatives to be considered throughout the rest of the survey. It read as follows: “For the following questions, we refer to meat alternatives as commercially available plant-based convenience foods that can be used instead of meat. Examples are vegetarian sausages, veggie patties, or plant-based minced ‘meat’”. The remaining questions (Q5–Q14) were then presented.

### 2.3. Data Analysis

First, we cleaned the data of obvious errors. During the data cleaning, 4 out of 550 individuals (0.7%) were removed from the dataset due to providing inconsistent or otherwise doubtful responses. Therefore, we included answers from 546 respondents in our further analyses.

Second, the composite scores for the published multi-item scales (Table 2) were calculated according to the instructions in the original sources [48,49,50,51,52,54]. Thanks to the use of an electronic questionnaire, the data included no missing values (i.e., no missed responses). Cleaned data (*N* = 546) with the calculated scores are available in the (Supplementary Materials Table S2).

The data were analyzed statistically using the IBM SPSS Statistics version 27 software package (IBM, Armonk, NY, USA). We applied descriptive and analytical statistics to the data, and we used α = 0.05 as the criterion for statistical significance. The independent samples *t*-test, one-way and two-way analysis of variance (ANOVA), and Pearson’s chi-squared tests were also used as appropriate. The answer categories “Daily” and “More than once per day” for the questions concerning the eating frequency of both meat and meat alternatives (Q5) were combined into one category named “Daily” to increase the clarity of the results. This category implies eating a food item at least once per day.

Essentially, we classified the respondents into six groups based on the hedonic tone (valence: negative–positive) of their first associations with meat (Q3) and meat alternatives (Q4), as described below (in Section 3.2). In this paper, we refer to these groups of respondents as (consumer) segments.

We employed a two-way ANOVA using the respondents’ gender and consumer segment as fixed factors (independent variables) in order to study the quantitative variables as appropriate. A full factorial model was run first and the significance of the gender×segment interaction was observed. If the interaction was nonsignificant, the interaction term was left out of the model and the results were reported based on the model including only the main effects. Furthermore, if the main effect of the segment was significant, Tukey’s post hoc test was applied to reveal which of the segments differed from the others.

## 3. Results

### 3.1. Demographics and Diet

The data (total *N* = 546) included more responses from women (322; 59.0%) than men (224; 41.0%). In comparison, according to official statistics concerning Finland [55], the gender distribution among 20–39-year-old Finns (at the end of 2019) was 48.4% women and 51.5% men [56]. 

The mean age of the respondents was 31.2 years and the age distribution was rather evenly distributed across 20–39 years (with the range defined by the inclusion criterion). The women respondents were, on average, a little younger than the men (30.6 vs. 32.0 years, respectively; *t*(504) = 3.04, *p* = 0.002). By contrast, the respondents’ education, as measured by the number of years (including both school and professional education), did not differ between the genders (16.3 vs. 15.9 years, respectively; *p* > 0.05).

Among all the respondents, about two-thirds (67.2%) identified themselves as omnivores (agreeing with the statement “I eat all animal products”), while about one-third (32.8%) followed a diet that limited the consumption of animal products in one way or other. Following a limited diet in terms of the consumption of meat/animal-based products was more prevalent among the women than the men. Indeed, nearly half of the women (42.5%) but only about a fifth of the men (18.8%) followed a non-omnivorous diet, that is, identified themselves as either flexitarian, pescetarian, vegetarian, or vegan (Pearson’s chi-square = 33.9, *p* < 0.001) (Table 3).

Furthermore, approximately two-thirds of the non-omnivores (66.5%, corresponding to 21.8% of all the respondents) were either flexitarians or pescetarians, while the remaining third of the non-omnivores (33.5%, corresponding to 11.0% of all the respondents) were either vegetarians or vegans.

### 3.2. Hedonic Tones of the First Associations with Meat and Meat Alternatives

The hedonic tone (valence on a scale ranging from −5, “extremely negative”, to 5, “extremely positive”) of the first associations (words, images, or thoughts) spontaneously evoked when thinking about meat was, on average, close to neutral (1.1). Likewise, the average hedonic tone of the first associations with meat alternatives was close to neutral (1.0). No statistically significant difference was observed between the values (*t*(1090) = 0.61; *p* = 0.542). However, the individual differences in the ratings of the hedonic tones were large (SD 3.4 and 3.1 for meat and meat alternatives, respectively), implying that not all the respondents rated their associations as neutral.

#### 3.2.1. Hedonic Tone by Diet and Gender

The two-way ANOVA involving diet and gender as fixed factors showed no significant diet×gender interaction in terms of the hedonic tone of the first associations with either meat (F(4,536) = 1.52; *p* = 0.195) or meat alternatives (F(4,536) = 0.75; *p* = 0.560). This implied that within a given diet group, both genders provided similar ratings.

Diet had a significant main effect on the hedonic tones of the first associations evoked by both meat (F(4,540) = 191.1; *p* < 0.001) and meat alternatives (F(4,540) = 44.1; *p* < 0.001). Similarly, gender had a significant main effect in the case of both meat (F(1,540) = 7.6; *p* = 0.006) and meat alternatives (F(1,540) = 5.9; *p* = 0.015). The omnivores and men rated their first associations with meat as more positive (and those with meat alternatives as more negative) than the non-omnivores (i.e., flexitarians, pescetarians, vegetarians, and vegans) and women, respectively (Table 4).

Diet appeared to more clearly influence the respondents’ hedonic responses to their first associations with meat than their first associations with meat alternatives. Although the overall means of the hedonic tones concerning meat and meat alternatives were similar, the difference between the means in the most extreme diet groups in terms of the hedonic tone associated with meat was 7.1 points (from −4.2 in vegans to 2.9 in omnivores), while it was only 4.0 points in the case of meat alternatives (from −0.1 in omnivores to 3.9 in vegans). Among the non-omnivorous diet groups, significant differences were observed in the average hedonic tone associated with meat but not that associated with meat alternatives (Table 4).

We observed a clear negative correlation between the hedonic tones associated with meat and meat alternatives, although the correlation was not strong (Pearson’s r = −0.55, *p* < 0.01). Among the omnivores (the largest diet group) in particular, there was wide variation in the hedonic tone associated with meat alternatives (SD 2.9), although the mean was close to zero (neutral). Some omnivores may have had positive associations with both meat and meat alternatives, or alternatively, they may have regarded both neutrally. This led us to assume that it could prove useful to classify the respondents into segments based on the hedonic tones associated with both meat and meat alternatives (instead of using the hedonic tone associated with either meat or meat alternatives).

#### 3.2.2. Segmentation of the Respondents

We cross-tabulated the ratings of the hedonic tones of the first associations with meat and meat alternatives to identify potential clusters of respondents that could be used as consumer segments in further analyses. Indeed, a visual inspection of the crosstab suggested that the hedonic responses were clustered, not evenly distributed.

**Figure 1:**
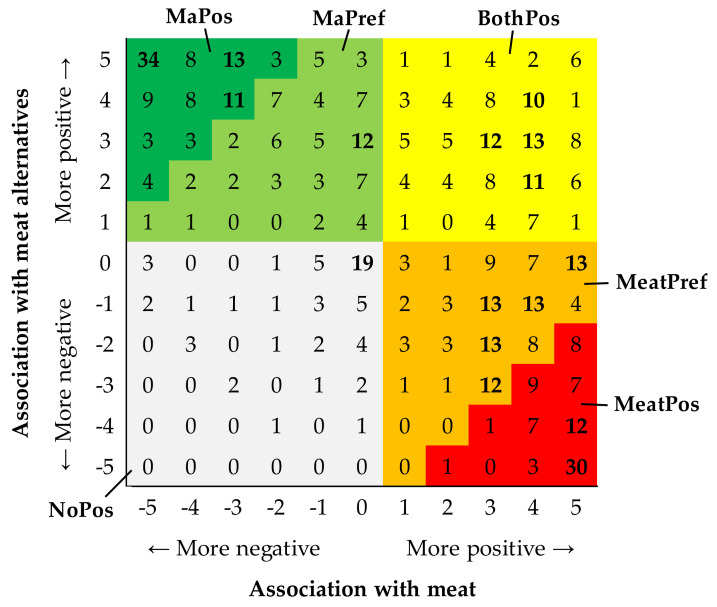
Cross-tabulation of the hedonic tones (valence, on a scale from −5 to 5) of the first associations evoked by meat and plant-based meat alternatives and classifying the respondents into six consumer segments (marked with different colors). The numbers in the cells denote the counts of individual respondents who gave the respective combination of responses. Counts ≥10 are marked in bold to highlight the clustering (total *N* = 546 individuals).

We identified six clusters, which we defined and labeled as follows: The most obvious clusters existed in the upper left corner of the crosstab (those respondents who had very positive associations with meat alternatives (Ma) but negative associations with meat, labeled “MaPos” and marked with dark green in Figure 1) and the lower right corner (those who had very positive associations with meat but negative associations with meat alternatives, labeled “MeatPos” and marked with red in Figure 1). Furthermore, between these two extreme clusters in the corners, there were groups of respondents who slightly or moderately preferred their associations with meat (labeled “MeatPref” and marked with orange in Figure 1) or meat alternatives (labeled “MaPref” and marked with light green in Figure 1). However, there was also a cluster of respondents who reported positive associations with both meat and meat alternatives (labeled “BothPos” and marked with yellow in Figure 1). Finally, there was a cluster of respondents who did not report positive associations with either meat or meat alternatives, instead rating the associations with both as neutral or even slightly negative (labeled “NoPos” and marked with light grey in Figure 1).

The definition, size, and gender distribution of the formed consumer segments are summarized in Table 5. The size of the segments ranged from 58 (10.6%) to 129 (23.6%) individuals. The percentage of women in a segment increased with an increasing preference for meat alternatives (Table 5). By contrast, no difference in age (F(5,540) = 1.6; *p* = 0.158) or number of years in education (F(5,540) = 1.2; *p* = 0.314) was observed between the segments.

The omnivores represented the largest fraction in all the segments, except for the segment most positive with regard to meat alternatives (MaPos). Unsurprisingly, the segments that reported the associations with meat to have relatively more positive hedonic tones (MeatPos and MeatPref) consisted almost exclusively of omnivores. Yet, more than half of the respondents in the segments that did not exhibit a clear difference in terms of the hedonic tones (BothPos and NoPos) were also omnivores. Moreover, the omnivores even represented the largest diet group in the segment that reported a higher hedonic tone with regard to meat alternatives (MaPref), although this segment also consisted of a remarkable fraction of flexitarians and pescetarians (Table 6). The dominance of the omnivores in almost all the segments can be explained by the fact that the omnivores were also the overall largest diet group (67.2% of all respondents).

### 3.3. Consumption Frequency of Meat and Meat Alternatives and the Underlying Reasons Why

#### 3.3.1. Consumption

**Figure 2:**
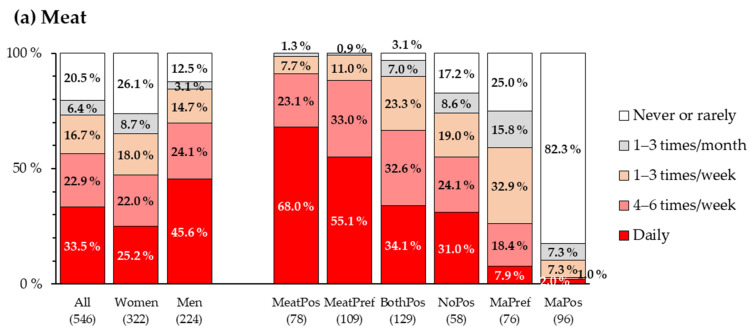

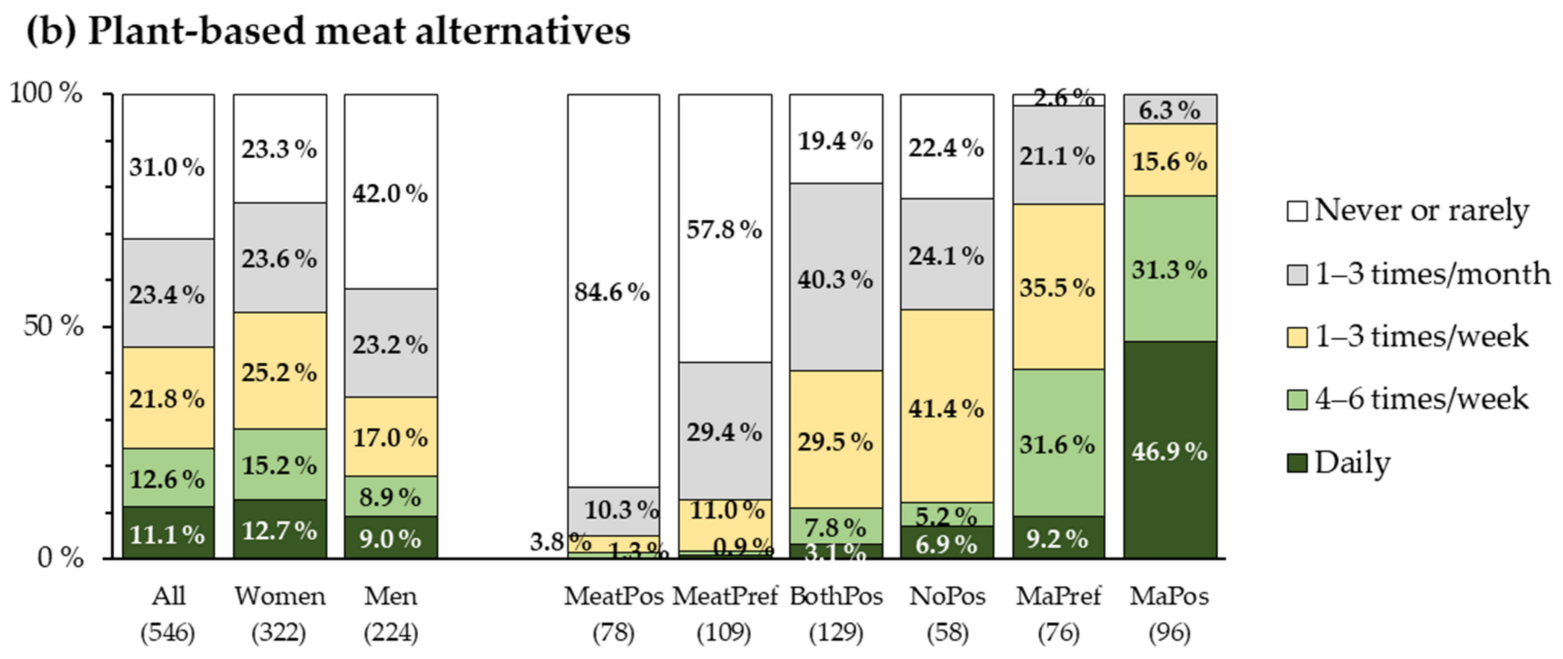
Use frequencies of (**a**) meat (pork, poultry, beef, ham, sausages, etc.) and (**b**) plant-based meat alternatives (including vegetarian patties, soy, tofu, etc.) by gender and consumer segment. The number of individuals in a group is given in parentheses. For details concerning how the respondents were classified into segments, see Figure 1 and Table 5.

Meat, including various meat products (but not fish), was consumed on a daily basis by a third of the respondents (33.5%). By contrast, a fifth (20.5%) of the respondents reported eating meat never or only rarely. Notably, the remainder, that is, almost half of the studied millennials (46.0%), reported sometimes eating meat but abstaining from it at least one day per week. As expected, the segments that reported their associations with meat to have a more positive hedonic tone (Table 5) also consumed meat more frequently (Figure 2a).

Plant-based meat alternatives were eaten daily by only about 11% of the respondents, although almost half of the respondents (45.5%) consumed them at least once a week. About two-thirds of the millennials (68.9%) ate meat alternatives at least once a month, whereas about one-third (31.0%) ate them rarely or never. As in the case of meat, the hedonic tone of the first associations with meat alternatives was reflected in how often such products were consumed (Figure 2b). These findings suggest that the hedonic tones of the first associations with meat and meat alternatives could be used to predict people’s consumption of these food categories.

Next, we asked how many respondents consumed both meat and meat alternatives. Some overlap in terms of the consumption of these foods was expected because, in the case of both meat and meat alternatives, the majority of respondents reported eating them at least occasionally. In addition, we expected that some respondents consumed meat alternatives in an attempt to reduce their meat consumption (while not totally abstaining from eating meat), as 12.3% identified themselves as flexitarians (Table 3) and almost a quarter (23.6%) reported positive hedonic tones with regard to the associations with both meat and meat alternatives (Table 5).

**Figure 3:**
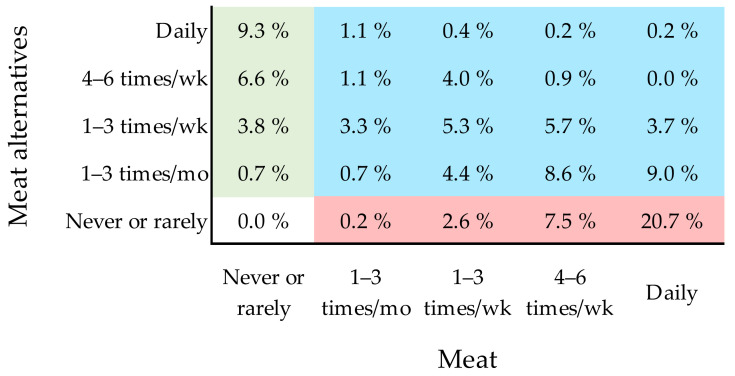
Cross-tabulation of the consumption frequencies of meat (pork, poultry, beef, ham, sausages, etc.) in columns and plant-based meat alternatives (including vegetarian patties, soy, tofu, etc.) in rows. The percentages in the cells denote the proportion of respondents who responded with the combination represented by that cell (out of the total *N* = 546 respondents). Among all the respondents, 31.0% (red cells) consumed only meat, 20.4% (green cells) consumed only meat alternatives, and 48.6% (blue cells) consumed both meat and meat alternatives.

To investigate this issue, we cross-tabulated the consumption frequencies of meat and meat alternatives. This confirmed that almost half of the respondents (48.6%) ate both meat and meat alternatives at least once a month. Only meat (no meat alternatives) was eaten by 31.0%, while only meat alternatives (no meat) were eaten by 20.4% of the respondents. Notably, about a fifth of the respondents (20.4%) regularly ate (at least once a week) both meat and meat alternatives (Figure 3). The consumer segment that reported positive associations with both meat and meat alternatives (BothPos) represented the largest group among those who consumed both meat and meat alternatives at least once a month (37.7%) and those who consumed them on a weekly basis (36.0%).

#### 3.3.2. Reasons for Eating and Not Eating Meat Alternatives

The question about why a respondent ate or did not eat plant-based meat alternatives was connected to a separate simple question concerning the consumption of meat alternatives. We first asked, “Do you eat plant-based meat alternatives?” (Q6), which had three response options. If the answer to Q6 was “Yes, on a regular basis”, we then asked, “Why do you eat plant-based meat alternatives regularly?” (Q7a). If the answer to Q6 was “No” or “I have sampled meat alternatives but do not eat them on a regular basis”, the next question was “Why do you not eat plant-based meat alternatives regularly?” (Q7b). Both questions concerning the reasons for eating/not eating meat alternatives were check-all-that-apply (CATA)-type questions with 7 (Q7a) and 12 (Q7b) predefined response options.

Approximately 4 out of 10 respondents (40.8%) reported eating plant-based meat alternatives on a regular basis. The regular consumption of meat alternatives was more common among the women (47.8%) than the men (30.8%) (Χ^2^_(2)_ = 17.6; *p* < 0.001). The proportion of regular users of meat alternatives varied widely across the consumer segments (from 2.6% for MeatPos to 92.7% for MaPos) (Table 7).

Environmental reasons were the most frequently cited motive for the regular consumption of meat alternatives among all the respondents (80.7%), followed by animal welfare reasons (64.6%) and health reasons (53.8%) (Table 8). There were some differences in motives between the genders. Notably, a larger proportion of women (59.7%) than men (33.3%) selected “I like the taste” as a reason for regularly eating meat alternatives.

The consumer segments differed in terms of their motives for eating meat alternatives. Environmental reasons were among the two most commonly mentioned reasons in all the segments, while they were the top motive for the MaPos, MaPref, and NoPos segments. Interestingly, the most frequently reported motive for the MeatPref and BothPos segments was “I like trying new foods”.

Among those respondents who did not consume meat alternatives regularly, the most commonly cited reason for this behavior was “I do not like the taste of meat alternatives” (56.7%), followed by “Meat alternatives are too expensive” (51.4%) (Table 9). These two reasons were the top two reasons given by both the women and the men. However, in terms of the women, the third most commonly mentioned reason for not eating meat alternatives regularly was “I do not know how to cook meat alternatives”, whereas for men it was “Meat alternatives are not a good replacement for meat”.

The main reasons for not eating meat alternatives regularly also differed among the consumer segments. For the segments that reported a less positive hedonic tone with regard to meat alternatives (Meat Pos and Meat Pref), the top reason was clearly “I do not like the taste of meat alternatives”. For the segments that reported a positive attitude toward meat alternatives (BothPos and MaPref) but who still do not eat such products regularly, the two most frequently mentioned reasons were “Meat alternatives are too expensive” and “I do not know how to cook meat alternatives”. While the frequency of citing various reasons varied considerably among the segments in general, the reason “Meat alternatives are too expensive” was mentioned by a somewhat similar proportion of individuals in all the segments (42.1–56.1%).

### 3.4. Status of Meat in Meals

#### 3.4.1. Importance of Meat in Main Meals

We asked the respondents “How important do you consider meat to be for your main meal in the following situations?”, that is, for a “typical weekday”, “weekend”, and “at a restaurant” (Q8, 7-point scale ranging from 1, “Not important at all”, to 7, “Very important”). The mean rating for the importance of meat in a main meal was close to the midpoint of the scale and similar for the typical weekday (3.6), weekend (3.9), and at a restaurant (4.0) options.

The women considered meat in all of the given situations to be less important than the men did (indicating the significant main effect of gender). The mean importance ratings given by the women and men were 3.1 vs. 4.4 for meat in a main meal on a typical weekday (F(1,539) = 29.7; *p* < 0.001), 3.4 vs. 4.6 on the weekend F(1,539) = 25.9; *p* < 0.001), and 3.4 vs. 4.8 at a restaurant F(1,539) = 17.6; *p* < 0.001), respectively.

The consumer segments varied greatly in terms of their responses here. The main effect of the segment was significant for meat on a typical weekday (F(5,539) = 121.9; *p* < 0.001), on the weekend (F(5,539) = 127.2; *p* < 0.001), and at a restaurant (F(5,539) = 118.9; *p* < 0.001). As expected, the MeatPos segment rated the importance of meat in all the studied situations the highest, while the MaPos segment rated it the lowest.

#### 3.4.2. Difficulty of Thinking of a Vegetarian Main Course for Invited Guests

The responses to the question “How difficult is it for you to think of a vegetarian main course for invited guests?” (Q9, rated on an 11-point scale from 0, “Very easy”, to 10, “Very difficult”) varied widely among the respondents. The women regarded it as easier to think of a vegetarian main course for guests than the men (2.8 vs. 5.0, indicating a significant main effect for gender (F(1,539) = 18.5; *p* < 0.001). Similarly, the consumer segment had a significant main effect on the responses to this question (F(5,539) = 58.2; *p* < 0.001). As expected, among the various segments, the MaPos segment rated it the easiest to think of a vegetarian main course for guests (0.3), followed by the MaPref (1.9), BothPos (3.5), NoPos (3.7), MeatPref (5.6), and MeatPos (7.5) segments (the means of all the segments, except those of the BothPos and NoPos segments, differed from each other according to Tukey’s test, *p* < 0.05).

### 3.5. Diet-Related Attitudes

Finally, we analyzed whether the genders and consumer segments differed in terms of their responses to the selected multi-item scales. All the scales showed good internal consistency as measured using Cronbach’s alpha: diet-related health consciousness (0.77), ecological welfare concerns (0.90), importance of the natural content of foods (0.90), meat commitment (0.97), and food neophobia (0.89).

The women scored higher than the men in relation to the Ecological Welfare Scale (3.2 vs. 2.8, F(1,539) = 16.2; *p* < 0.001) and Natural Content Scale (2.8 vs. 2.6, F(1,539) = 22.1; *p* < 0.001). By contrast, the women scored lower than the men in terms of the Meat Commitment Scale (2.8 vs. 4.2, F(1,539) = 37.3; *p* < 0.001). No significant main effect of gender was observed with regard to scores for Health Consciousness Scale or Food Neophobia Scale (Table 10).

According to the two-way ANOVA, the consumer segment had a significant main effect on the scores for all the attitude scales: Health Consciousness (F(5,539) = 6.6; *p* < 0.001), Ecological Welfare (F(5,539) = 19.3; *p* < 0.001), Natural Content (F(5,539) = 4.0; *p* = 0.001), Meat Commitment (F(5,539) = 179.3; *p* < 0.001), and Food Neophobia (F(5,539) = 3.5; *p* = 0.004). Tukey’s test confirmed these results and classified the segments into different homogeneous subsets for all the variables except food neophobia. In the case of food neophobia, Tukey’s test classified all the segments into the same homogeneous subset (*p* = 0.058) (Table 10).

The differences between the segments were the most obvious when it came to meat commitment and ecological welfare: the MaPos and MaPref segments were less committed to meat and more concerned about ecological welfare than the MeatPos and MeatPref segments. The scores from the scales measuring health consciousness, naturalness, and food neophobia did not reveal any systematic differences across the segments.

## 4. Discussion

### 4.1. Hedonic Tones of the Associations with Meat and Meat Alternatives

The millennials’ mean hedonic tones evoked by the first associations with meat and meat alternatives were similar and slightly positive (1.1 vs. 1.0, respectively, on a scale ranging from −5 to 5). This finding conflicts somewhat with the findings of the studies reviewed by Onwezen et al. [23], who concluded that acceptance of alternative proteins was relatively low when compared with acceptance of meat. Our finding that the millennials’ associations with meat alternatives were, on average, as positive as their associations with meat may reflect millennials’ greater concern and knowledge regarding environmental issues when compared with older generations [33,34]. The millennials’ orientation toward plant-based diets was also supported by the higher proportion of vegans and vegetarians in the present study (total 11.0%) when compared with the general population of 25–74-year-old Finns in 2017 (1.8%) [31] and 18–79-year old Finns in 2018 (6.7%) [46].

As expected, the women reported, on average, more positive associations with plant-based meat alternatives (and less positive associations with meat) than the men. However, the women rated their associations with meat alternatives as more positive than those with meat (1.5 vs. 0.4), which suggests that millennial women are, at least in countries such as Finland, a potential target group for plant-based meat alternative products.

It was also expected that the followers of diets that limited the consumption of meat (i.e., flexitarians, pescetarians, vegetarians, and vegans) would report negative associations with meat and positive associations with meat alternatives. Our findings confirmed that the vegetarians and vegans reported positive associations with meat alternatives more frequently, as did the flexitarians, although some plant-based meat alternatives on the market may resemble meat closely (to appeal to flexitarians). Interestingly, only 3.4% of those who did not regularly eat meat alternatives mentioned “Meat alternatives are too much like meat” as a reason why. This suggests that the plant-based meat alternative products sold in Finland do not resemble meat to such an extent that vegetarians are put off.

The individual variation in the hedonic tones of the associations with meat and meat alternatives was wide, which formed the basis for our segmentation procedure. As meat alternatives are by definition designed to replace meat in a person’s diet, we assumed that it would be useful to study attitudes toward both meat and meat alternatives (not only toward one of them). Indeed, when cross-tabulating the ratings for the hedonic value of the associations with meat vs. meat alternatives, we observed that the ratings for meat and meat alternatives were not always simply opposite values (positive association with meat combined with negative association with meat alternatives, or vice versa), as some respondents reported positive (or neutral) associations with both meat and meat alternatives.

### 4.2. Consumption of Meat and Meat Alternatives and the Underlying Reasons Why

Both gender and consumer segment were associated with the consumption of meat and meat alternatives. The women and the consumer segments that reported more positive associations with meat alternatives (MaPref and MaPos) ate meat alternatives more frequently than the men and the consumer segments that reported more positive associations with meat (MeatPref and MeatPos). The men’s greater preference for meat was in line with the findings of prior research [46,57,58]. In Finland, according to the National FinDiet 2017 Survey [59], even 79% of men but only 26% of women ate more red and processed meat than the national guidelines recommend (500 g/week [60]).

The consumption of meat did not exclude the consumption of meat alternatives. This observation is consistent with the finding by Götze and Brunner [45] that meat alternatives can serve as a complementary component in one’s diet. In a survey by Smart Protein project conducted in 2021 in adult consumers of 10 European countries (Austria, Denmark, France, Germany, Italy, Netherlands, Poland, Romania, Spain, UK), on average, 30% of the respondents identified themselves as flexitarians [24]. In the present study, around half of the respondents (48.6%) ate both meat and meat alternatives at least once per month. Thus, they can be referred to as flexitarians in a broad sense. However, a much lower proportion of respondents (12.3%) actually identified themselves as flexitarians. This could be because the description of a flexitarian in our questionnaire was strict (“I only rarely eat meat”) and because the concept of flexitarian may still be largely unfamiliar to the general public. Nevertheless, the fraction of regular users of meat alternatives and those who had at least tried them totaled 84.0% in the present study (mean age 31.2 years). A slightly lower percentage (76%) of somewhat older respondents (mean age 57.4 years) had tried meat alternatives in the study by Götze and Brunner [45].

Environmental reasons represented the top motive for eating meat alternatives regularly. Among the women (and the respondents overall), the second most commonly cited motive was animal welfare, whereas among the men it was health reasons. A similar set of reasons, that is, “ecological welfare” and “health” (together with “sensory appeal”), were found to be the top food choice motives for using meat substitutes in the study by Hoek et al. [43]. These results suggest that many consumers regard meat alternatives as healthy. However, the nutritional value of novel plant-based meat alternatives may not always be as high as thought, because some products can, for example, contain high amounts of saturated fat and sodium. For further discussion on nutritional aspects of meat alternatives, see the review by Tso et al. [61] and commentary by Tso and Forde [62]. Of course, the quality of the diet as a whole is more important than its single items, also when considering replacement of animal-based foods in a diet [63]. For example, results from a clinical intervention study by Päivärinta et al. [64] indicated that replacing part of the animal-based proteins with plant-based proteins in a Nordic diet increased fiber intake, improved fat quality, and benefited blood lipoprotein profile.

In the present study, the drivers of consumption differed between the consumer segments, similar to the situation in previous studies [43,65]. Interestingly, for the MeatPref and BothPos segments, the most frequently given reason for regularly eating meat alternatives was “I like trying new foods”. Although food neophobia (i.e., reluctance to try new foods) has been frequently identified as one of the barriers to the consumption of alternative proteins [23,46,66], it may be more important in relation to certain other kinds of meat alternatives, such as insects and cultured meat, than plant-based meat alternatives [23]. Moreover, in the present study, the consumer segments did not differ significantly (according to Tukey’s test) in terms of their Food Neophobia Scale scores.

With regard to the barriers to consumption, the most commonly cited reasons for not eating meat alternatives regularly were “I do not like the taste of meat alternatives” and “Meat alternatives are too expensive”. Taste being given as a reason for not eating meat alternatives is consistent with previous findings by, for example, Hoek et al. [43] (for a review, see [67]). Similarly, price was identified as a top barrier toward eating plant-based products in flexitarians in the survey by Smart Protein project [24]. Likewise, price being given as a barrier is in line with the conclusion by Michel et al. [20] that meat alternatives must be offered at competitive prices if they are to have a good chance of replacing meat. However, the frequencies of citing reasons differed considerably between the consumer segments. For example, among those who did not use meat alternatives regularly despite reporting relatively positive associations with them (from the MaPref and BothPos segments), one of the most frequently mentioned reasons for nonuse was “I do not know how to cook meat alternatives”.

### 4.3. Consumer Segments

In contrast to the present study, Lemken et al. [42] and Niva and Vainio [46] used latent class analysis and Götze and Brunner [45] hierarchical cluster analysis and multiple variables to segment consumers from different countries (Germany/New Zealand, Finland, and Switzerland, respectively) and studied adults of all ages. Despite the clear differences between these studies and the present investigation, they all ended up with a similar number of segments (5–6/population) with comparable features. All four studies identified a consumer cluster firmly oriented toward eating meat. Lemken et al. [42] termed the consumer group resembling our “MeatPos” segment the “meat only” cluster; Niva and Vainio [46], “established beef lovers”; and Götze and Brunner [45], the “uncompromising meat-eaters”. Similarly, all the authors identified a segment strongly devoted to meat alternatives/legumes. The majority of individuals in the former type of segment were men, while the majority in the latter were women [42,45,46].

Most respondents in the present study (68.1%), similar to the situation in the studies by Lemken et al. (55.7% in Germany and 57.3% in New Zealand) [42], Niva and Vainio (53.8%) [46], and Götze and Brunner (67.6%) [45], were classified into the middle groups/segments, whose attitudes toward meat/meat alternatives and/or their consumption were not as extreme as those in the two segments described above. The middle segments arguably exhibit the highest potential to reduce their meat consumption by replacing it with meat alternatives. The segments with the strongest orientation toward meat may prove resistant to interventions intended to reduce meat consumption, while the segments that report the strongest avoidance of meat may not need to reduce their consumption. Therefore, the middle segments could be the best targets for interventions aiming to reduce meat consumption with the help of plant-based meat alternatives.

### 4.4. Limitations

The present study focused on millennials (20–39-year-old individuals). No respondents from other age groups were studied. Thus, we cannot directly compare millennials to consumers from other generations in the same population. Likewise, we only included respondents from one country/culture (Finland) in our study. However, we compared our results with those of relevant prior studies conducted in other countries and with wider age ranges of respondents [42,45]. Furthermore, we have allowed access not only to our results but also to our questionnaire (Table S1) and data (Table S2) to enable other researchers to utilize them in future studies.

Most of the questions in our survey were derived from published and validated multi-item scales (Table 2). However, among the scales, a validated translation was only available in Finnish for the Food Neophobia Scale [68]. Nevertheless, four of the present authors, who were all native Finnish speakers and experts in the field of food sciences, proofread the translations of the other scales. Yet, we acknowledge the need for further validation of these scales in the Finnish language and culture.

## 5. Conclusions

Our survey data, which were obtained from a representative sample of Finnish millennials, suggest that the hedonic tones of the first associations with meat vs. plant-based meat alternatives (positive-negative) are not unidimensional; rather, they are two-dimensional phenomena that can be used for easy consumer segmentation. The hedonic tone associated with meat alternatives was opposite to that associated with meat for some respondents, albeit not for all of them. In fact, some people think positively about both meat and meat alternatives, while other consumers are neutral concerning both food categories. Our classification of consumers was performed based on their responses to two simple questions, and it led to six segments. This allowed us to distinguish not only people who exclusively promote meat or vegetarian diets but also those who have positive attitudes toward both meat and meat alternatives. These respondents were mostly flexitarians or omnivores who consumed meat alternatives because they liked to try new foods, in addition to environmental reasons. Thus, this consumer segment was considered the best target group for behavioral interventions designed to replace meat consumption with the consumption of meat alternatives.

## Figures and Tables

**Table 1:** Survey questions 1–9: specific questions on diet, education, hedonic tone, consumption of meat and meat alternatives, reasons for use/nonuse, and importance of meat in meals and for guests.

No.	Question ^1^	Response Options
Q1	Diet	Omnivore; Flexitarian; Pescetarian; Vegetarian; Vegan
Q2	Education in years ^2^	(Number of years)
Q3 ^3^	Hedonic tone (valence) of the first association with meat	11-point scale from “Extremely negative” (−5) to “Extremely positive” (+5)
Q4 ^3^	Hedonic tone (valence) of the first association with meat alternatives	11-point scale from “Extremely negative” (−5) to “Extremely positive” (+5)
Q5	“How frequently do you eat (1) meat (pork, poultry, beef, ham, sausages, etc.) and (2) meat alternatives?”	Never or rarely; 1–3 times per month; 1–3 times per week; 4–6 times per week; Daily; More than once per day
Q6	“Do you eat plant-based meat alternatives?”	“Yes, on a regular basis”; “I have sampled meat alternatives, but do not eat them on a regular basis”; “No”
Q7a	“Why do you eat plant-based meat alternatives regularly?” (only if the response to Q6 was “Yes, on a regular basis”)	Check all that apply from among 8 options (including an “Other reason” option)
Q7b	“Why do you not eat plant-based meat alternatives regularly?” (only if the response to Q6 was other than “Yes, on a regular basis”)	Check all that apply from among 12 options
Q8	“How important do you consider meat to be for your main meal in the following situations?” (1) Typical weekday; (2) Weekend; (3) Restaurant	7-point scale from “Not important at all” (1) to “Very important” (7)
Q9	“How difficult is it for you to think of a vegetarian main course for invited guests?”	11-point scale from “Very easy” (0) to “Very difficult” (10)

^1^ The Finnish translation of these questions is available in the (Supplementary Materials Table S1). ^2^ Education was the only demographical factor probed in the survey. The age and gender of the respondents were available from the register of the utilized market research company. ^3^ The order of presentation of Q3 and Q4 was randomized.

**Table 2:** Survey questions 10–14: multi-item scales.

No.	Scale ^1^	No. of Items	Example of the Items	Response Options	Reference
Q10	Diet-Related Health Consciousness Scale	4	“I think it is important to eat healthily.”	7-point Likert scale from “Do not agree at all” (1) to “Totally agree” (7) ^2^	Dohle et al., 2014 [48] ^3^
Q11	Ecological Welfare Scale	5	“It is important that the food I eat on a typical day…”, e.g., “…has been produced in a way that animals have not experienced pain.”	Not at all important (1); A little important (2); Moderately important (3); Very important (4)	Lindeman and Väänänen, 1999 [49] ^4^
Q12	Natural Content Scale	4	“It is important that the food I eat on a typical day…”, e.g., “… contains no additives.”	Not at all important (1); A little important (2); Moderately important (3); Very important (4)	Steptoe et al., 1995 [50] ^5^
Q13	Meat Commitment Scale	7	“I don’t want to eat meals without meat.”	7-point Likert scale from “Strongly disagree” (1) to “Strongly agree” (7)	Piazza et al., 2015 [51]
Q14	Food Neophobia Scale	10	“I don’t trust new foods.”	7-point Likert scale from “Strongly disagree” (1) to “Strongly agree” (7)	Pliner and Hobden, 1992 [52]

^1^ The Finnish translation of these questions is available in the (Supplementary Materials Table S1). ^2^ A seven-point scale was used instead of the original six-point scale (from “Don’t agree at all” [1] to “Fully agree” [6]) used by Dohle et al. [48]. ^3^ The Diet-Related Health Consciousness Scale by Dohle et al. [48] was partly based on the items from the Health Consciousness Scale by Schifferstein and Oude Ouphuis [53]. ^4^ One of the three scales developed by Lindeman and Väänänen [49], namely the Ecological Welfare Scale (including the subscales for Animal Welfare and Environment Protection), was used in this study. ^5^ The original three-item Natural Content Scale (part of the Food Choice Questionnaire) was complemented with a fourth item, “…is as little processed as possible”.

**Table 3:** Respondents’ diet by gender.

Diet	All		Women		Men	
	*n*	%	*n*	%	*n*	%
Omnivore	367	67.2	185	57.5	182	81.3
Flexitarian	67	12.3	52	16.1	15	6.7
Pescetarian	52	9.5	41	12.7	11	4.9
Vegetarian	25	4.6	19	5.9	6	2.7
Vegan	35	6.4	25	7.8	10	4.5
Total	546	100.0	322	100.0	224	100.0

**Table 4:** Hedonic tones of first associations with meat and plant-based meat alternatives (rated on a scale from −5 to 5) by diet and gender.

Group		Meat		Meat Alternatives	
	*N*	Mean	SD	Mean	SD
Diet ^1^					
Omnivore	367	2.9 d	2.1	−0.1 a	2.9
Flexitarian	67	−1.0 c	2.7	2.8 b	2.3
Pescetarian	52	−2.8 b	2.0	3.4 b	1.9
Vegetarian	25	−3.8 ab	1.6	3.5 b	2.1
Vegan	35	−4.2 a	1.9	3.9 b	1.5
Gender					
Women	322	0.4	3.5	1.5	3.0
Men	224	2.1	3.0	0.2	3.0
All	546	1.1	3.4	1.0	3.1

^1^ The means among the diet groups (within a column) not sharing a common letter are significantly different (Tukey’s test, *p* < 0.05).

**Table 5:** Consumer segments based on the hedonic tones of the first associations with meat and plant-based meat alternatives.

Segment	Definition	Women ^1^	Men ^1^	Total	Of All ^2^
MeatPos	Hedonic tone with meat was ≥7 points higher than with meat alternatives.	33 42.3%	45 57.7%	78 100.0%	14.3%
MeatPref	Hedonic tone with meat was positive (and 3–6 points higher than with meat alternatives), while it was negative with meat alternatives.	53 48.6%	56 51.4%	109 100.0%	20.0%
BothPos	Hedonic tone was positive with both meat and meat alternatives.	74 57.4%	55 42.6%	129 100.0%	23.6%
NoPos	Hedonic tone was neutral or negative with both meat and meat alternatives.	37 63.8%	21 36.2%	58 100.0%	10.6%
MaPref	Hedonic tone with meat alternatives was positive (and 3–6 points higher than with meat), while it was negative with meat.	50 65.8%	26 34.2%	76 100.0%	13.9%
MaPos	Hedonic tone with meat alternatives was ≥7 points higher than with meat.	75 78.1%	21 21.9%	96 100.0%	17.6%

^1^ Values of the prevailing gender in a segment are highlighted in bold. ^2^ Relative size of a segment out of all 546 respondents.

**Table 6:** Diet by consumer segment.

Segment ^1^	Including ^2^				
	Omnivore	Flexitarian	Pescetarian	Vegetarian	Vegan
MeatPos	77 98.7%	1 1.3%	0 0.0%	0 0.0%	0 0.0%
MeatPref	106 97.2%	2 1.8%	1 0.9%	0 0.0%	0 0.0%
BothPos	110 85.3%	15 11.6%	2 1.6%	0 0.0%	2 1.6%
NoPos	38 65.5%	9 15.5%	7 12.1%	3 5.2%	1 1.7%
MaPref	32 42.1%	24 31.6%	12 15.8%	6 7.9%	2 2.6%
MaPos	4 4.2%	16 16.7%	30 31.3%	16 16.7%	30 31.3%

^1^ Consumer segments formed based on the hedonic tones of the first associations with meat and plant-based meat alternatives (see Figure 1 and Table 5). ^2^ Values of the largest diet group in a segment are highlighted in bold. Note that the majority of all respondents (67.2%) were omnivores.

**Table 7:** Overall consumption of plant-based meat alternatives by gender and consumer segment.

“Do You Eat Plant-Based Meat Alternatives?” ^1^	All (546) ^2^	Women (322)	Men (224)	Consumer Segment					
				Meat-Pos (78)	Meat-Pref (109)	Both-Pos (129)	NoPos (58)	MaPref (76)	MaPos (96)
Yes, on a regular basis	40.8%	47.8%	30.8%	2.6%	10.1%	37.2%	27.6%	75.0%	92.7%
I have sampled meat alternatives but do not eat them on a regular basis	43.2%	39.8%	48.2%	42.3%	67.9%	53.5%	60.3%	23.7%	7.3%
No	15.9%	12.4%	21.0%	55.1%	22.0%	9.3%	12.1%	1.3%	0.0%

^1^ The percentages (%) within a column indicate the proportion of a group who responded with a given answer. The most common response within each group is highlighted in bold. ^2^ The total number of individuals in a group is given in parentheses.

**Table 8:** Reasons for eating plant-based meat alternatives regularly: percentage of regular eaters (40.8% of all respondents) offering a specific reason by gender and consumer segment.

“Why Do You Eat Plant-Based Meat Alternatives Regularly?” ^1^	All (223) ^2^	Women (154)	Men (69)	Consumer Segment					
				MeatPos (2)	Meat-Pref (11)	BothPos (48)	NoPos (16)	MaPref (57)	MaPos (89)
Because…									
of environmental reasons	80.7%	84.4%	72.5%	n/a ^3^	54.5%	66.7%	81.3%	80.7%	91.0%
of animal welfare reasons	64.6%	69.5%	53.6%	n/a	27.3%	37.5%	50.0%	64.9%	86.5%
of health reasons	53.8%	52.6%	56.5%	n/a	36.4%	47.9%	43.8%	54.4%	59.6%
I like the taste	51.6%	59.7%	33.3%	n/a	0.0%	41.7%	37.5%	61.4%	59.6%
I like trying new foods	50.2%	51.3%	47.8%	n/a	72.7%	75.0%	43.8%	54.4%	31.5%
my social environment expects me to eat meat alternatives	11.2%	11.7%	10.1%	n/a	27.3%	18.8%	12.5%	8.8%	5.6%
of financial reasons	7.2%	7.1%	7.2%	n/a	9.1%	6.3%	12.5%	1.8%	9.0%
other	4.9%	3.9%	7.2%	n/a	9.1%	4.2%	12.5%	5.3%	3.4%

^1^ The percentages within a column indicate the proportion of a group who responded with a given answer (multiple answers possible). The response options were sorted from the most to the least frequent response among all the respondents. The two most frequent responses within each group are highlighted in bold. ^2^ The total number of regular eaters in a group is given in parentheses. ^3^ n/a, not applicable. Because only 2 out of 78 (2.6%) respondents in the MeatPos segment ate meat alternatives regularly, their responses are not shown.

**Table 9:** Reasons for not eating plant-based meat alternatives regularly: percentage of those who did not eat meat alternatives regularly (59.2% of all respondents) offering a specific reason by gender and consumer segment.

“Why Do You Not Eat Plant-Based Meat Alternatives Regularly?” ^1^	All (323) ^2^	Women (168)	Men (155)	Consumer Segment					
				MeatPos (76)	Meat-Pref (98)	BothPos (81)	NoPos (42)	MaPref (19)	MaPos (7)
I do not like the taste of meat alternatives	56.7%	47.6%	66.5%	75.0%	70.4%	38.3%	50.0%	15.8%	n/a ^3^
Meat alternatives are too expensive	51.4%	48.2%	54.8%	42.1%	56.1%	53.1%	52.4%	47.4%	n/a
Meat alternatives are too processed	37.8%	41.7%	33.5%	56.6%	37.8%	21.0%	40.5%	31.6%	n/a
I do not know how to cook meat alternatives	34.1%	44.6%	22.6%	10.5%	33.7%	49.4%	33.3%	57.9%	n/a
Meat alternatives are not a good replacement for meat	31.6%	22.0%	41.9%	61.8%	37.8%	12.3%	19.0%	0.0%	n/a
My family won’t eat it	22.3%	29.8%	14.2%	21.1%	23.5%	27.2%	19.0%	10.5%	n/a
Meat alternatives are unhealthy	11.8%	9.5%	14.2%	30.3%	10.2%	1.2%	9.5%	0.0%	n/a
Meat alternatives are something for vegans and vegetarians only	10.2%	6.0%	14.8%	22.4%	11.2%	2.5%	7.1%	0.0%	n/a
Meat alternatives are too much packaged	9.9%	11.9%	7.7%	17.1%	4.1%	11.1%	7.1%	10.5%	n/a
Meat alternatives are not available where I go shopping	6.5%	6.5%	6.5%	3.9%	8.2%	4.9%	7.1%	10.5%	n/a
I do not know what meat alternatives are	5.6%	5.4%	5.8%	2.6%	9.2%	4.9%	4.8%	5.3%	n/a
Meat alternatives are too much like meat	3.4%	2.4%	4.5%	2.6%	3.1%	1.2%	7.1%	10.5%	n/a

^1^ The percentages within a column indicate the proportion of a group who responded with a given answer (multiple answers possible). The response options were sorted from the most to the least frequent response among all the respondents. The two most frequent responses within each group are highlighted in bold. ^2^ The total number of regular eaters in a group is given in parentheses. ^3^ n/a, not applicable. Because only 7 out of 96 (7.3%) respondents in the VegePos segment did not eat meat alternatives regularly, their responses are not shown.

**Table 10:** Scores for the diet-related attitude scales by gender and consumer segment (means (M) and standard deviations (SD)).

Scale (Potential Range) [Reference]		All (546) ^1^	Women (322)	Men (224)	Consumer Segment ^3^					
					MeatPos (78)	Meat-Pref (109)	BothPos (129)	NoPos (58)	MaPref (76)	MaPos (96)
Health Consciousness (1–7) [48]	M	5.2	5.2	5.2	5.1 ab	4.9 a	5.2 ab	5.1 ab	5.4 bc	5.7 c
	SD	1.1	1.0	1.1	1.3	1.0	1.0	1.1	1.0	1.0
Ecological Welfare (1–4) [49]	M	3.0	3.2 ^2^	2.8 ^2^	2.7 a	2.7 ab	3.0 abc	3.0 bc	3.2 c	3.6 d
	SD	0.7	0.7	0.8	0.8	0.7	0.7	0.8	0.6	0.4
Natural Content (1–4) [50]	M	2.7	2.8 ^2^	2.6 ^2^	2.9 b	2.7 ab	2.7 ab	2.8 ab	2.7 ab	2.5 a
	SD	0.8	0.8	0.8	0.9	0.8	0.8	0.8	0.8	0.8
Meat Commitment (1–7) [51]	M	3.4	2.8 ^2^	4.2 ^2^	6.2 f	4.7 e	3.4 d	2.9 c	1.8 b	1.1 a
	SD	2.1	1.9	2.0	1.0	1.5	1.4	1.7	1.0	0.4
Food Neophobia (10–70) [52]	M	28.8	28.7	28.9	31.7 a	30.5 a	26.8 a	31.2 a	27.6 a	26.9 a
	SD	11.5	11.8	11.0	12.6	11.8	11.2	12.9	10.1	9.8

^1^ The total number of individuals in a group is given in parentheses. ^2^ The main effect of gender was significant for these variables (ANOVA, *p* < 0.05). ^3^ The main effect of segment was significant for all the variables (ANOVA, *p* < 0.05). The letters denote results of the Tukey’s test, that is, which of the mean values (segments) are statistically different and which not (in a given variable, i.e., within a line). Lowest mean value has been marked with “a”, next lowest with “b” and so on. The means between the segments (within the same row) not sharing a common lowercase letter differed (Tukey’s test, *p* < 0.05).

## Data Availability

The data that have been analyzed for this article (anonymized responses to the online survey) are available as (Supplementary Materials Table S2).

## Supplementary Materials

The following supporting information can be downloaded at: https://www.mdpi.com/article/10.3390/foods11030456/s1, Table S1: Survey questions (in English and Finnish), Table S2: Data (responses to the online survey).
