# Supplementary material for: Millennials’ Consumption of and Attitudes toward Meat and Plant-Based Meat Alternatives by Consumer Segment in Finland
Source: Foods. 2022 Feb 3;11(3):456. doi: 10.3390/foods11030456 (PMC8834568; doi:10.3390/foods11030456)
Supplement: Supplementary file 1 [file foods-11-00456-s001.zip › TableS1.pdf]

Table S1. Survey questions (in English and Finnish). Values in parentheses next to the response options corresponds values in the data (Table S2).

No. [1]	Description [2]	English (original) [3]	Finnish (translation) [3]
Q1	Diet	Which of the following terms describes your diet best?<br>(1) Omnivore (I eat all animal products)<br>(2) Pescetarian (I do not eat meat, but fish and seafood)<br>(3) Flexitarian (I only rarely eat meat)<br>(4) Vegetarian (I do not eat meat and fish)<br>(5) Vegan (I do not eat any animal products)	Mikä seuraavista termeistä kuvaa parhaiten noudattamaasi ruokavaliota?<br>(1) Kaikkiruokainen (Syön kaikkia eläintuotteita)<br>(2) Pescovegetaristi (En syö lihaa, mutta syön kalaa ja mereneläviä)<br>(3) Fleksaaja (Syön vain harvoin lihaa)<br>(4) Kasvissyöjä (En syö lihaa enkä kalaa)<br>(5) Vegaani (En syö mitään eläintuotteita)
Q2	Education (in years)	How many years of education (school and professional education) have you had?	Kuinka monta vuotta olet opiskellut yhteensä (koulussa ja ammatillisesti)?
Q3 [4]	Hedonic tone (valence) of the first association to meat	When you think about **meat**, what are your first associations (words, pictures or thoughts) that spontaneously come to mind?<br>When you think about your first association, does it evoke a positive or a negative feeling?<br>(-5) Extremely negative<br>(-4)<br>(-3)<br>(-2)<br>(-1)<br>(0)<br>(1)<br>(2)<br>(3)<br>(4)<br>(5) Extremely positive	Kun ajattelet **lihaa**, mitkä ovat ensimmäiset mielikuvat (sanat, kuvat tai ajatukset), jotka tulevat spontaanisti mieleesi?<br>Kun ajattelet ensimmäistä mieleesi tullutta mielikuvaa, herättääkö se sinussa positiivisen vai negatiivisen tunteen?<br>(-5) Äärimmäisen negatiivinen<br>(-4)<br>(-3)<br>(-2)<br>(-1)<br>(0)<br>(1)<br>(2)<br>(3)<br>(4)<br>(5) Äärimmäisen positiivinen

Q4 [4]	Hedonic tone (valence) of the first association to meat alternatives	When you think about **meat alternatives**, what are your first associations (words, pictures or thoughts) that spontaneously come to mind?<br><br>When you think about your first association, does it evoke a positive or a negative feeling?<br><br>(-5) Extremely negative<br>(-4)<br>(-3)<br>(-2)<br>(-1)<br>(0)<br>(1)<br>(2)<br>(3)<br>(4)<br>(5) Extremely positive	Kun ajattelet lihan tapaan käytettäviä **kasviproteiinituotteita**, mitkä ovat ensimmäiset mielikuvat (sanat, kuvat tai ajatukset), jotka tulevat mieleesi?<br><br>Kun ajattelet ensimmäistä mieleesi tullutta mielikuvaa, herättääkö se sinussa positiivisen vai negatiivisen tunteen?<br><br>(-5) Äärimmäisen negatiivinen<br>(-4)<br>(-3)<br>(-2)<br>(-1)<br>(0)<br>(1)<br>(2)<br>(3)<br>(4)<br>(5) Äärimmäisen positiivinen
Q5	Consumption frequency of meat and meat alternatives	How frequently do you eat<br><br>(I) meat (pork, poultry, beef, ham, sausages etc.)?<br><br>(II) meat alternatives (including vegetarian patties, soy, tofu etc.)?<br><br>(1) Never or rarely<br>(2) 1-3 times per month<br>(3) 1-3 times per week<br>(4) 4-6 times per week<br>(5) Daily<br>(6) More than once per day	Kuinka usein syöt<br><br>(I) lihaa (sianlihaa, siipikarjaa, nautaa, kinkkua, makkaroita jne.)?<br><br>(II) Kasviproteiinituotteita (sisältäen kasvispihvit, soijan, tofun jne.)<br><br>(1) En koskaan tai harvoin<br>(2) 1-3 kertaa kuukaudessa<br>(3) 1-3 kertaa viikossa<br>(4) 4-6 kertaa viikossa<br>(5) Päivittäin<br>(6) Useammin kuin kerran päivässä
Q6	Overall consumption of meat alternatives	Do you eat meat alternatives?<br><br>(1) Yes, on a regular basis<br>(2) I have sampled meat alternatives, but do not eat them on a regular basis<br>(3) No	Syötkö lihan tapaan käytettäviä kasviproteiinituotteita?<br><br>(1) Kyllä, säännöllisesti<br>(2) Olen kokeillut kasviproteiinituotteita, mutta en syö niitä säännöllisesti<br>(3) En

Q7a	Reasons for consuming meat alternatives<br><br>(Only if the response to the Q6 was “Yes, on a regular basis”)	Why are you consuming meat alternatives?<br><br>Because ...<br><br>(Multiple answers possible)<br><br>• of health reasons<br>• of environmental reasons<br>• of financial reasons<br>• of animal welfare reasons<br>• I like the taste<br>• my social environment expects me to eat meat alternatives<br>• I like trying new foods<br>• other	Miksi syöt lihan tapaan käytettäviä kasviproteiinituotteita säännöllisesti?<br><br>(Voit valita useamman vaihtoehdon.)<br><br>• Terveydellisistä syistä<br>• Ympäristöön liittyvistä syistä<br>• Taloudellisista syistä<br>• Eläinten hyvinvoinnin vuoksi<br>• Pidän niiden mausta<br>• Sosiaalinen ympäristöni odottaa minun syövän kasviproteiinituotteita<br>• Pidän uusien ruokien kokeilusta<br>• Muu syy
Q7b	Reasons for not consuming meat alternatives<br><br>(Only if the response to the Q6 was other than “Yes, on a regular basis”)	Why do you not, or no longer, consume meat alternatives?<br><br>Because ...<br><br>(Multiple answers possible)<br><br>• I do not know what meat alternatives are<br>• Meat alternatives are too expensive<br>• I do not like the taste of meat alternatives<br>• My family won't eat it<br>• Meat alternatives are something for vegans and vegetarians only<br>• Meat alternatives are too much like meat<br>• Meat alternatives are not a good replacement for meat<br>• I do not know how to cook meat alternatives<br>• Meat alternatives are too much packaged<br>• Meat alternatives are too processed<br>• Meat alternatives are unhealthy<br>• Meat alternatives are not available where I go shopping	Miksi et syö kasviproteiinituotteita säännöllisesti?<br><br>Koska…<br><br>(Voit valita useamman vaihtoehdon)<br><br>• En tiedä mitä kasviproteiinituotteet ovat.<br>• Kasviproteiinituotteet ovat liian kalliita.<br>• En pidä kasviproteiinituotteiden mausta.<br>• Perheeni ei söisi kasviproteiinituotteita.<br>• Kasviproteiinituotteet on tarkoitettu vain vegaaneille ja kasvissyöjille.<br>• Kasviproteiinituotteet muistuttavat liian paljon lihaa.<br>• Kasviproteiinituotteet eivät ole hyvä vaihtoehto lihalle.<br>• En tiedä, miten laittaisin kasviproteiinituotteista ruokaa.<br>• Kasviproteiinituotteet ovat liian pakattuja.<br>• Kasviproteiinituotteet ovat liian prosessoituja.<br>• Kasviproteiinituotteet ovat epäterveellisiä.

			• Kasviproteiinituotteita ei ole tarjolla siellä, missä käyn kaupassa.
Q8	Importance of meat in meals	How important do you consider **meat** to be for your main meal in the following situations?<br>(I) Meat in a main meal on a typical weekday.<br>(II) Meat in a main meal on the weekend.<br>(III) Meat in a main meal eaten at a restaurant.<br>(1) Not important at all<br>(2)<br>(3)<br>(4)<br>(5)<br>(6)<br>(7) Very important	Kuinka tärkeänä pidät **lihaa** osana pääruokaasi seuraavissa tilanteissa?<br>(I) Liha pääruuassa tyypillisenä arkipäivänä.<br>(II) Liha pääruuassa viikonloppuna.<br>(III) Liha pääruuassa ruokailtaessa ravintolassa.<br>(1) Ei lainkaan tärkeä<br>(2)<br>(3)<br>(4)<br>(5)<br>(6)<br>(7) Erittäin tärkeä
Q9	Difficulty of thinking of a vegetarian main course for guests	How difficult is it for you to think of a vegetarian main course for invited guests?<br>(0) Very easy<br>(1)<br>(2)<br>(3)<br>(4)<br>(5)<br>(6)<br>(7)<br>(8)<br>(9)<br>(10) Very difficult	Kuinka vaikeaa sinusta on ajatella tarjoavasi kasvisruokaa pääruokana kutsutuille vieraillesi?<br>(0) Erittäin helppoa<br>(1)<br>(2)<br>(3)<br>(4)<br>(5)<br>(6)<br>(7)<br>(8)<br>(9)<br>(10) Very difficult

Q10	Diet-Related Health Consciousness Scale [5]<br><br>(Dohle et al., 2014) [1]	Please indicate your level of agreement for the following statements.<br><br>(I) I think it is important to eat healthily<br><br>(II) My health is dependent on how and what I eat.<br><br>(III) If one eats healthily, one gets ill less frequently.<br><br>(IV) I am prepared to leave a lot of food to eat as healthily as possible.<br><br>(1) Do not agree at all<br><br>(2)<br><br>(3)<br><br>(4)<br><br>(5)<br><br>(6)<br><br>(7) Totally agree	Ota kantaa, kuinka samaa mieltä olet seuraavista väittämistä.<br><br>(I) Mielestäni on tärkeää syödä terveellisesti.<br><br>(II) Terveyteni on riippuvainen siitä, miten ja mitä syön.<br><br>(III) Jos syö terveellisesti, myös sairastuu harvemmin.<br><br>(IV) Olen valmis olemaan syömättä monia ruokia, jotta voisin syödä niin terveellisesti kuin mahdollista.<br><br>(1) En lainkaan samaa mieltä<br><br>(2)<br><br>(3)<br><br>(4)<br><br>(5)<br><br>(6)<br><br>(7) Täysin samaa mieltä
Q11	Ecological Welfare Scale [6]<br><br>(Lindeman and Väänänen, 1999) [2]	Please indicate how important you perceive the following statements to be for you.<br><br>It is important that the food I eat on a typical day...<br><br>(I) ... has been produced in a way that animals have not experienced pain.<br><br>(II) ... has been produced in a way that animals' rights have been respected.<br><br>(III) ... has been prepared in an environmentally friendly way.<br><br>(IV) ... has been produced in a way which has not shaken the balance of nature.<br><br>(V) ... is packaged in an environmentally friendly way.<br><br>(1) Not at all important<br><br>(2) A little important<br><br>(3) Moderately important<br><br>(4) Very important	Valitse vaihtoehto, joka kuvaa parhaiten seuraavien väittämien tärkeyttä sinulle.<br><br>Minulle on tärkeää, että ruoka jota syön tavallisena päivänä…<br><br>(I) …on tuotettu niin, että eläimet eivät ole kokeneet kipua.<br><br>(II) …on tuotettu niin, että eläinten oikeuksia on kunnioitettu.<br><br>(III) …on valmistettu ympäristöystävällisesti.<br><br>(IV) …on valmistettu horjuttamatta luonnon tasapainoa.<br><br>(V) …on pakattu ympäristöystävällisesti.<br><br>Ei lainkaan tärkeää<br><br>(1) Ei lainkaan tärkeää<br><br>(2) Hieman tärkeää<br><br>(3) Melko tärkeää<br><br>(4) Erittäin tärkeää

Q12	Natural Content Scale [7]<br><br>(a part of the Food Choice Questionnaire; Steptoe et al., 1995) [3]	Please indicate how important the following statements are for you.<br><br>It is important that the food I eat on a typical day...<br><br>(I) ... contains no additives<br><br>(II) ... contains natural ingredients<br><br>(III) ... contains no artificial ingredients<br><br>(IV) ... is as little processed as possible<br><br>(1) Not at all important<br><br>(2) A little important<br><br>(3) Moderately important<br><br>(4) Very important	Valitse vaihtoehto, joka kuvaa parhaiten seuraavien väittämien tärkeyttä sinulle.<br><br>Minulle on tärkeää, että ruoka, jota syön tavallisena päivänä…<br><br>(I) …ei sisällä lisäaineita.<br><br>(II) …sisältää luonnollisia raaka-aineita.<br><br>(III) …ei sisällä keinotekoisia raaka-aineita.<br><br>(IV) …on mahdollisimman vähän prosessoitu.<br><br>(1) Ei lainkaan tärkeää<br><br>(2) Hieman tärkeää<br><br>(3) Melko tärkeää<br><br>(4) Erittäin tärkeää
Q13	Meat Commitment Scale<br><br>(Piazza et al., 2015) [4]	Please indicate your level of agreement for the following statements.<br><br>(I) I don't want to eat meals without meat.<br><br>(II) When choosing food, I virtually always select the meat option.<br><br>(III) I can't imagine giving up meat.<br><br>(IV) I am committed to eating meat.<br><br>(V) The best part of most meals is the meat portion.<br><br>(VI) I would never give up eating meat.<br><br>(VII) I cannot imagine substituting meat from a meal.<br><br>(1) Strongly disagree<br><br>(2)<br><br>(3)<br><br>(4) Neither agree nor disagree<br><br>(5)<br><br>(6)<br><br>(7) Strongly agree	Valitse seuraaviin väittämiin vaihtoehto, joka parhaiten kuvaa mielipidettäsi.<br><br>(I) En halua syödä aterioita ilman lihaa.<br><br>(II) Kun valitsen ruokaa, niin valitsen lähes aina lihavaihtoehdon.<br><br>(III) En voi kuvitella luopuvani lihasta.<br><br>(IV) Olen sitoutunut syömään lihaa.<br><br>(V) Paras osa useimmista aterioista on lihan osuus.<br><br>(VI) En koskaan luopuisi lihan syömisestä.<br><br>(VII) En voi kuvitella korvaavani lihaa ateriasta.<br><br>(1) Täysin eri mieltä<br><br>(2)<br><br>(3)<br><br>(4) Ei samaa eikä eri mieltä<br><br>(5)<br><br>(6)<br><br>(7) Täysin samaa mieltä

Q14	Food Neophobia Scale [8]<br><br>(Pliner and Hobden, 1992) [5]	Please indicate your level of agreement for the following statements.<br><br>(I) I am constantly sampling new and different foods.<br><br>(II) I don't trust new foods.<br><br>(III) If I don't know what is in a food, I won't try it.<br><br>(IV) I like foods from different countries.<br><br>(V) Ethnic food looks too weird to eat.<br><br>(VI) At dinner parties, I will try a new food.<br><br>(VII) I am afraid to eat things I have never had before.<br><br>(VIII) I am very particular about the foods I will eat.<br><br>(IX) I will eat almost anything.<br><br>(X) I like to try new ethnic restaurants.<br><br>(1) Strongly disagree<br><br>(2)<br><br>(3)<br><br>(4) Neither agree nor disagree<br><br>(5)<br><br>(6)<br><br>(7) Strongly agree	Valitse seuraaviin väittämiin vaihtoehto, joka parhaiten kuvaa mielipidettäsi.<br><br>(I) Kokeilen jatkuvasti uusia ja erilaisia ruokia.<br><br>(II) En luota uusiin ruokiin.<br><br>(III) Ellen tiedä, mitä ruoka sisältää, en kokeile sitä.<br><br>(IV) Pidän eri maiden ruoista.<br><br>(V) Muiden maiden ruoat näyttävät liian oudoilta syötäviksi.<br><br>(VI) Kokeilen uusia ruokia, kun olen ruokavieraana.<br><br>(VII) Minua epäilyttää syödä ruokia, joita en ole ennen kokeillut.<br><br>(VIII) Olen hyvin valikoiva siinä, mitä ruokia syön.<br><br>(IX) Syön melkein mitä tahansa.<br><br>(X) Minusta on hauskaa kokeilla eri maiden ruokakulttuureja edustavia ravintoloita.<br><br>(1) Täysin eri mieltä<br><br>(2)<br><br>(3)<br><br>(4) Ei samaa eikä eri mieltä<br><br>(5)<br><br>(6)<br><br>(7) Täysin samaa mieltä

[1] Number of the question refer to the numbers of the questions in Table 1 and 2, and variables in Table S2 (Data). The numbers do not indicate the order of the questions in the survey. The survey included also other questions not used for this article.
[2] Description added to the table to help reading (this text was not included in the survey).
[3] Texts for questions and response options. The options were given only once if the same were used for all items in a scale. Individual items in multi-item questions/scales are marked in Roman numerals (I, II, etc.). The values in parentheses next to the response options corresponding the values in the data (Table S2) were not shown to the respondents on the online form except for the numerical rating scales in Q3, Q4, and Q8–Q14.
[4] The order of presentation of questions 3 and 4 was randomized.
[5] Diet-Related Health Consciousness scale by Dohle et al. was based partly on the items of Health Consciousness Scale by Schifferstein and Oude Ouphuis [6].
[6] One of the three scales developed by Lindeman and Väänänen, the Ecological Walfare Scale (including subscales for Animal Welfare and Environment Protection), was included in this study.
[7] The original 3-item Natural Content scale (part of the Food Choice Questionnaire) was com-plemented with fourth item, "…is as little processed as possible."
[8] The wording of half of the statements is reversed related to food neophobia. Therefore, scoring of these statements must be reversed before calculating the score (for details, see Pliner and Hobden [5]).

References

1. Dohle, S.; Hartmann, C.; Keller, C. Physical Activity as a Moderator of the Association between Emotional Eating and BMI: Evidence from the Swiss Food Panel. *Psychol. Health* **2014**, *29*, 1062–1080, doi:10.1080/08870446.2014.909042.
2. Lindeman, M.; Väänänen, M. Measurement of Ethical Food Choice Motives. *Appetite* **2000**, *34*, 55–59, doi:10.1006/appe.1999.0293.
3. Steptoe, A.; Pollard, T.M.; Wardle, J. Development of a Measure of the Motives Underlying the Selection of Food: The Food Choice Questionnaire. *Appetite* **1995**, *25*, 267–284, doi:10.1006/appe.1995.0061.
4. Piazza, J.; Ruby, M.B.; Loughnan, S.; Luong, M.; Kulik, J.; Watkins, H.M.; Seigerman, M. Rationalizing Meat Consumption. The 4Ns. *Appetite* **2015**, *91*, 114–128, doi:10.1016/j.appet.2015.04.011.
5. Pliner, P.; Hobden, K. Development of a Scale to Measure the Trait of Food Neophobia in Humans. *Appetite* **1992**, *19*, 105–120, doi:10.1016/0195-6663(92)90014-W.
6. Schifferstein, H.; Oude Ophuis, P. Health-Related Determinants of Organic Food Consumption in The Netherlands. *Food Qual. Prefer.* **1998**, *9*, 119–133, doi:10.1016/S0950-3293(97)00044-X.
